# Pseudoxanthoma Elasticum With Comedones in a 12-Year-Old Female Patient: A Case Report

**DOI:** 10.7759/cureus.47041

**Published:** 2023-10-14

**Authors:** Rawan Almutairi, Humoud Al-Sabah

**Affiliations:** 1 Dermatology Department, Al-Sabah Hospital, Kuwait City, KWT

**Keywords:** histology, abcc6, dermatology, comedones, pseudo-pseudoxanthoma elasticum

## Abstract

Pseudoxanthoma elasticum (PXE) is a rare multisystem disease characterized by progressive calcification and disintegration of elastic fibers. The disorder is attributed to a genetic mutation occurring in the ABCC6 gene, which encodes for the ATP-binding cassette transporter C6. This gene is located on chromosome 16. Patients commonly present with cutaneous, ophthalmic, and cardiovascular manifestations. However, there is a significant degree of phenotypic diversity. The diagnosis is determined by clinical manifestations, histological analysis of the lesions, and genetic analysis. The present study includes a case report of a 12-year-old female patient who presented with a chief complaint of painless, mildly pruritic yellow papules located on her neck for a period of one year. These papules were accompanied by comedones.

## Introduction

PXE is a rare autosomal recessive disease with a 2:1 female preponderance and no racial or ethnic preferences. It is distinguished by progressive mineralization and fragmentation of elastic fibers in the skin, retina, gastrointestinal tract, and cardiovascular system. The disease results from a mutation in the ABCC6 gene mapped to chromosome 16 [1]. The prevalence of PXE is approximately one in 25.000 individuals; some people with milder phenotypes are likely to go undiagnosed [2]. This is a case report of a 12-year-old female patient with a one-year history of painless, mildly itchy yellow papules in her neck, along with comedones.

## Case presentation

**Figure 1:**
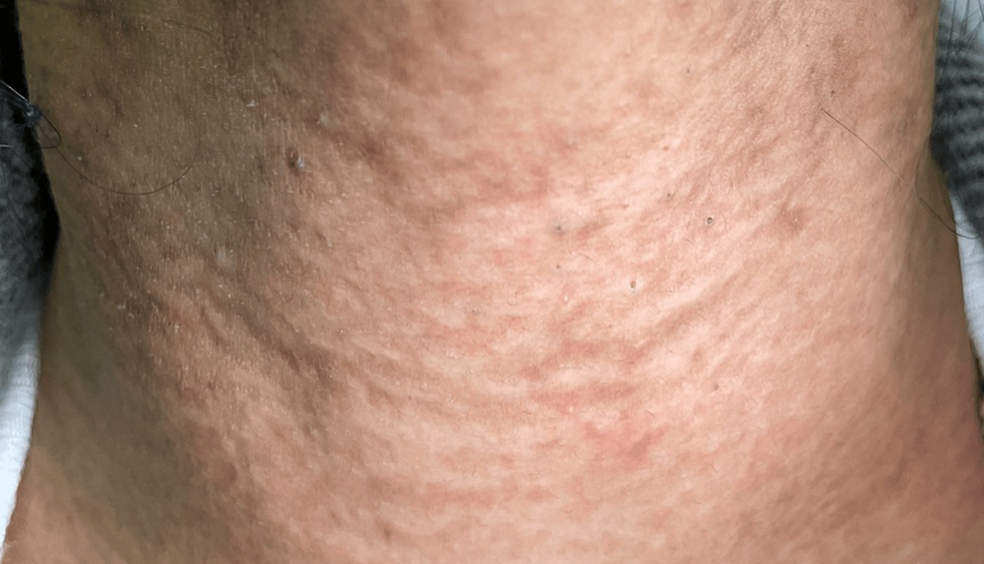
Pseudoxanthoma elasticum with comedones

**Figure 2:**
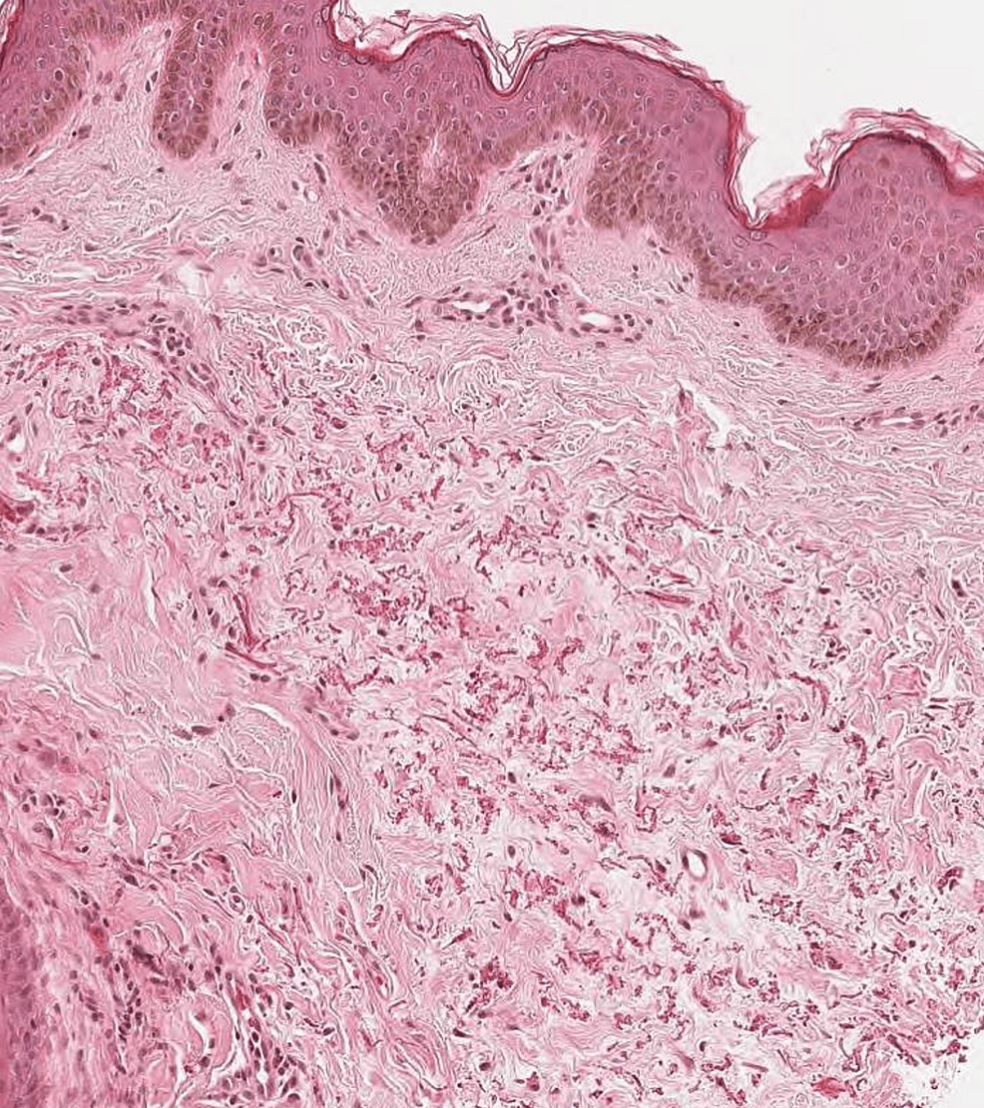
Histopathological features of pseudoxanthoma elasticum Hematoxylin-eosin-stained section shows altered elastic fibers throughout the dermis that are short, thick, irregularly clumped, and basophilic.

**Figure 3:**
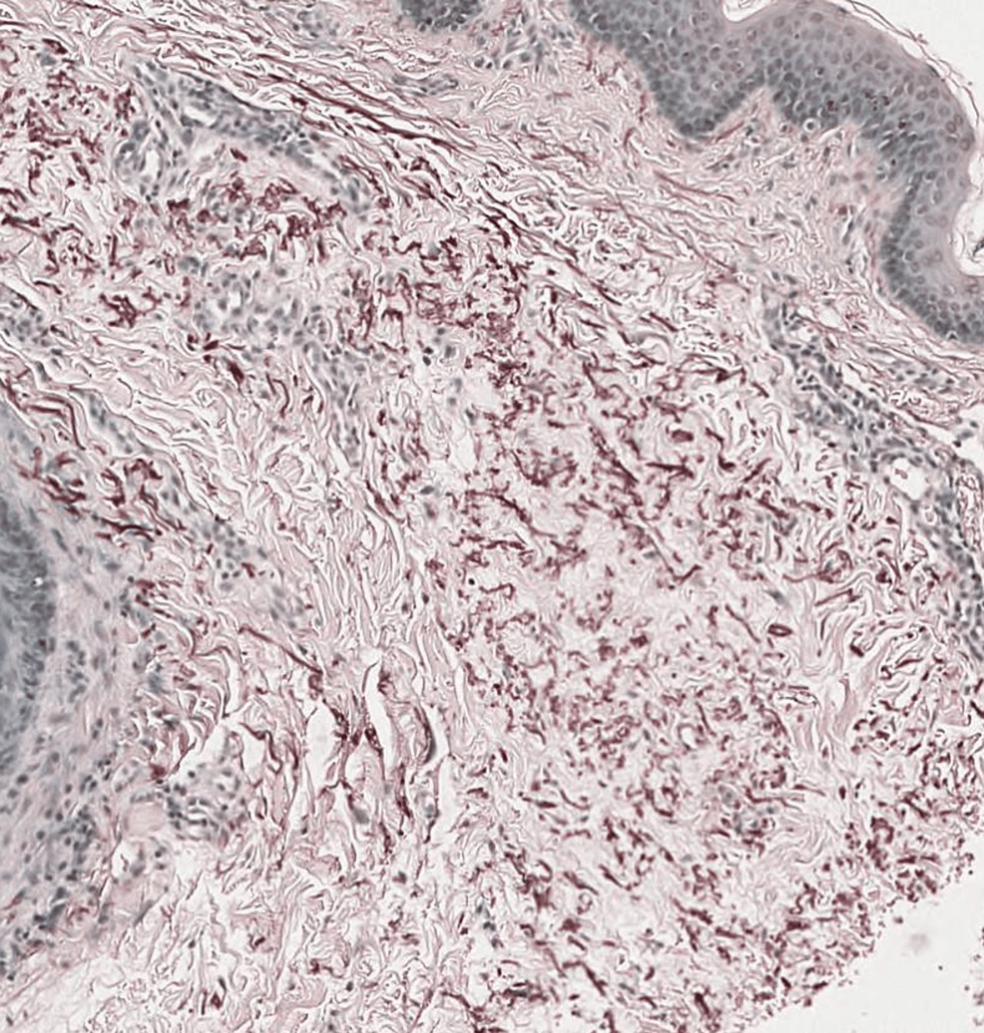
Histopathological features of pseudoxanthoma elasticum Elastic Van Gieson stain revealed clumped, fragmented elastic fibers in the mid-dermis.

**Figure 4:**
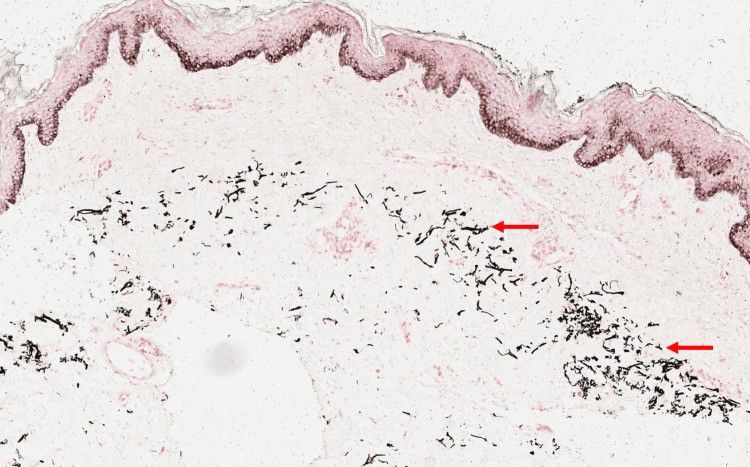
Histopathological features of pseudoxanthoma elasticum Von Kossa stain showed calcium deposits in the mid-dermis (red arrows).

A 12-year-old female patient presented to the dermatology clinic with a one-year history of multiple yellowish papules with dark discoloration over her neck that appeared gradually. The lesions were painless and slightly itchy. In the last three months, she has noticed the presence of black comedones. Previously, she had been a healthy child with no history of angina or visual disturbances. The patient had no relevant personal or family medical history of dermatosis. Upon examination, the patient's vital signs were normal. There were yellow papules arranged in a linear or reticular configuration, with intervening normal skin areas in between and the presence of some hyperpigmented areas. The lesions were present only in the anterior and lateral neck regions. A few open black comedones are presented (Figure 1). No other abnormalities were found during the rest of the physical examination. Laboratory investigations showed normal results. A biopsy specimen was taken for histopathological examination, which showed calcified elastic fibers in the mid-dermis using Hematoxylin and Eosin stain (Figure 2) and Elastic Van Gieson stain (Figure 3) and deposited calcium in Von Kossa stain (Figure 4).

The study was completed with a fundus examination that showed no abnormalities. The electrocardiogram and echocardiogram were both normal. Based on the clinical aspects of the lesions and histopathological findings, which were highly characteristic of PXE, the dermatopathologist consultant decided that there was no need to perform a study of the ABCC6 genes.

## Discussion

In most cases, the skin is the first organ system affected by PXE, leading to a diagnosis. The mean age at the first evident changes on the sides of the neck was 13 years. However, the average age of patients diagnosed with the disease was 22 years, with a nine-year delay between the onset of symptoms and confirmation of the diagnosis [3]. Our patient was classified as having early-onset PXE due to her young age and limited skin involvement, which was confined to her neck without any systemic symptoms.

Loss-of-function mutations in the ABCC6 gene, which primarily expresses the putative efflux transporter ABCC6 in the liver, are the primary cause of PXE. The pathogenesis of PXE has also been linked to decreased circulating levels of pyrophosphate, a potent mineralization inhibitor [4].

In PXE, the body's elastic tissue becomes mineralized, and calcium is deposited in the tissue. This can lead to alterations in the skin, eyes, cardiovascular system, and digestive system. An early sign of PXE is the appearance of small, 1-5 mm, asymptomatic, yellowish-soft papules presenting in a reticular pattern [5], as seen in our patient. Later in the progression of the disease, these lesions typically affect lower flexural areas, such as the axillae, groin, and back of the knee. Lesions can also coalesce into plaques that give the skin a "gooseflesh" or "plucked appearance. However, clinically visible skin changes are not pathognomonic for PXE, as comparable skin changes can also be seen in beta-thalassemia or Paget's disease [3].

In addition to cutaneous manifestations, PXE can affect the ophthalmic, cardiovascular, and gastrointestinal systems. Angioid retinal streaks, which range in color from slate gray to reddish-brown, are characteristic ocular manifestations of PXE. It is also possible for the retina to bleed and scar, leading to vision loss [6]. A range of cardiovascular signs and symptoms can be seen in PXE as a result of the level of blood vessel narrowing due to calcification of the elastic layer. Although rare, they include hypertension, arteriosclerosis, intermittent claudication, valvular, and coronary artery disease [7,8]. PXE also causes gastrointestinal hemorrhage in approximately 13% of affected individuals owing to arterial elastic fiber degeneration in the mucosa of the stomach and intestines [9].

The most significant histological characteristic of PXE is elastorrhexis, a pattern in the middle dermis characterized by progressive mineralization and disintegration of elastic fibers. Fragmented elastic fibers and mid-dermal calcification are both necessary for the histological diagnosis of PXE [10].

The multiple comedones seen in our patient are an uncommonly described characteristic of PXE in the medical literature. Comedone formation within classic PXE lesions is thought to be related to UV-induced degeneration of elastic fibers in the dermis, as observed in solar elastosis [11]. This was supported by the distribution of comedones on sun-exposed sites, such as the lateral neck, and their sparse distribution in adjacent sun-protected areas, such as the submental region [12]. This case illustrates the significance of diligent sun protection in averting comedone formation and accelerating elastic degeneration in PXE patients.

Owing to the systemic manifestations of pseudoxanthoma elasticum, its treatment requires a multidisciplinary approach. PXE is presently incurable but has a favorable prognosis if multidisciplinary teams follow up appropriately [13]. Adenovirus-mediated ABCC6 gene therapy demonstrates promise for the treatment of PXE in preclinical studies [4]. In childhood and adolescence, excessive calcium intake should be avoided, as a correlation between high calcium intake and PXE severity has been postulated [14]. Early diagnosis is essential for the proper management of associated complications, and treatment options primarily focus on symptom management and addressing complications.

## Conclusions

PXE is an uncommon genetic disorder characterized by the mineralization of elastic fibers in different tissues. This disease is caused by ABCC6 gene mutations. Multiple comedones, as observed in our patient, were atypical symptoms of PXE. Early diagnosis is crucial for the appropriate management of associated complications.

## References

[1] Marconi B, Bobyr I, Campanati A (2015). Pseudoxanthoma elasticum and skin: Clinical manifestations, histopathology, pathomechanism, perspectives of treatment. Intractable Rare Dis Res.

[2] Chassaing N, Martin L, Calvas P, Le Bert M, Hovnanian A (2005). Pseudoxanthoma elasticum: a clinical, pathophysiological and genetic update including 11 novel ABCC6 mutations. J Med Genet.

[3] Finger RP, Charbel Issa P, Ladewig MS, Götting C, Szliska C, Scholl HP, Holz FG (2009). Pseudoxanthoma elasticum: genetics, clinical manifestations and therapeutic approaches. Surv Ophthalmol.

[4] Huang J, Snook AE, Uitto J, Li Q (2019). Adenovirus-mediated abcc6 gene therapy for heritable ectopic mineralization disorders. J Invest Dermatol.

[5] Sakata S, Su JC, Robertson S, Yin M, Chow C (2006). Varied presentations of pseudoxanthoma elasticum in a family. J Paediatr Child Health.

[6] Zineb K (2018). Ophthalmologic manifestations of pseudoxanthoma elasticum. Oman J Ophthalmol.

[7] Campens L, Vanakker OM, Trachet B (2013). Characterization of cardiovascular involvement in pseudoxanthoma elasticum families. Arterioscler Thromb Vasc Biol.

[8] Ono H, Oshita A, Inaba S (2022). Pseudoxanthoma elasticum resulting in acute coronary syndrome. J Cardiol Cases.

[9] Qian SS, Kesar V, Shah F, Park D, Sorrentino D (2020). Pseudoxanthoma elasticum. ACG Case Rep J.

[10] Sasso BM, Cintra ML, de Souza EM (2017). Pseudoxanthoma elasticum. Autops Case Rep.

[11] Yeh C, Schwartz RA (2022). Favre-Racouchot disease: protective effect of solar elastosis. Arch Dermatol Res.

[12] Maarouf M, Sharon VR, Sivamani RK (2017). Familial pseudoxanthoma elasticum associated with multiple comedones. Dermatology online journal.

[13] Lucas C, Aranha J, da Rocha I, Sousa D (2020). Case report: Pseudoxanthoma elasticum. F1000Res.

[14] Hamamoto Y, Nagai K, Yasui H Hyperreactivity of pseudoxanthoma elasticum-affected dermis to vitamin D3. J Am Acad Dermatol2000.

